# The Dynamic Interplay Between Puberty and Structural Brain Development as a Predictor of Mental Health Difficulties in Adolescence: A Systematic Review

**DOI:** 10.1016/j.biopsych.2024.06.012

**Published:** 2024-10-01

**Authors:** Svenja Kretzer, Andrew J. Lawrence, Rebecca Pollard, Xuemei Ma, Pei Jung Chen, Nare Amasi-Hartoonian, Carmine Pariante, Corentin Vallée, Michael Meaney, Paola Dazzan

**Affiliations:** a Department of Psychological Medicine, Institute of Psychiatry, Psychology, and Neuroscience, King’s College London, London, United Kingdom; b Singapore Institute for Clinical Sciences, Agency for Science, Technology & Research (A∗STAR) Singapore, Republic of Singapore; c Department of Psychiatry, Chang Gung Memorial Hospital, Taoyuan, Taiwan; d NIHR Maudsley Biomedical Research Centre at South London and Maudsley NHS Foundation Trust and King’s College London, London, United Kingdom; f Douglas Hospital Research Centre, Department of Psychiatry, McGill University, Montreal, Quebec, Canada

**Keywords:** Adolescence, Brain structure, Mental health, Precision psychiatry, Puberty, Risk prediction

## Abstract

Puberty is a time of intense reorganization of brain structure and a high-risk period for the onset of mental health problems, with variations in pubertal timing and tempo intensifying this risk. We conducted 2 systematic reviews of articles published up to February 1, 2024, focusing on 1) the role of brain structure in the relationship between puberty and mental health, and 2) precision psychiatry research evaluating the utility of puberty in making individualized predictions of mental health outcomes in young people. The first review provides inconsistent evidence about whether and how pubertal and psychopathological processes may interact in relation to brain development. While most studies found an association between early puberty and mental health difficulties in adolescents, evidence on whether brain structure mediates this relationship is mixed. The pituitary gland was found to be associated with mental health status during this time, possibly through its central role in regulating puberty and its function in the hypothalamic-pituitary-gonadal and hypothalamic-pituitary-adrenal axes. In the second review, the design of studies that have explored puberty in predictive models did not allow for a quantification of its predictive power. However, when puberty was evaluated through physically observable characteristics rather than hormonal measures, it was more commonly identified as a predictor of depression, anxiety, and suicidality in adolescence. Social processes may be more relevant than biological ones to the link between puberty and mental health problems and represent an important target for educational strategies.

Adolescence, defined as the developmental period between childhood and adulthood, is a sensitive time for emerging mental health problems (1,2). Approximately 50% of adult mental disorders have an onset before the age of 15 years, and 75% start before the age of 18 years (3). Puberty relates to the biological processes around developing reproductive capability that usually start around the beginning of adolescence. It is marked by elaborate hormonal changes that induce development of fertility, secondary sex characteristics, and the adolescent growth spurt (4). Puberty might interact with the intense reorganization of brain structure during adolescence to create a particularly sensitive period for the emergence of mental health difficulties (5).

Pubertal development is preceded by adrenarche, the activation of the adrenal glands that initiate a rise in the steroid hormone dehydroepiandrosterone (DHEA) and its sulfate, DHEA-S (6). The hypothalamic-pituitary-gonadal (HPG) axis, which initiates hormonal cascades that produce steroid hormones, primarily drives later pubertal development (6) and interacts with the hypothalamic-pituitary-adrenal (HPA) axis, which secretes cortisol and DHEA (7). It has been suggested that pronounced activation of the entire endocrine system observed during puberty, including the HPG and HPA axes and changing thyroid hormone levels (8), increase vulnerability to mental health problems (7,9). Surges in steroid hormones induce physical maturation and changes in brain structure and function (10,11), such as the region-specific changes in dendritic spine density and myelination that underlie decreases in cortical volume and thickness and increases in cortical surface area (12,13). Deviations from normative brain developmental patterns during puberty have in turn been associated with the emergence of mental health problems (14,15). Subcortical and limbic structures such as the hippocampus, amygdala, or pituitary gland are highly affected by pubertal hormones and play a role in their secretion, and structural changes in these regions have been related to affective disorders and psychosis (12,16,17).

The substantial interindividual variability in healthy pubertal timing (variability in pubertal stage at any given age) (18) and tempo (speed of development) (19) have profound implications for adolescent mental health (5). Early pubertal timing has been associated with increased risk of internalizing symptoms, eating disorders, and substance abuse (20,21), and faster progression of development could be related to depressive symptoms (22). As in the conceptual model proposed by Pfeifer and Allen (5), puberty may be linked to adolescent-onset internalizing disorders via social and brain developmental processes evolving over adolescence. Children who begin puberty earlier or develop faster than their peers may experience difficulties adapting to concomitant physical changes and intensified social risks, such as bullying and social pressure. In turn, pubertal hormones acting early on relatively immature brain structures may provide the neurobiological substrate that explains the link between early timing of puberty and subsequent mental health difficulties (6). For example, early pubertal timing has been associated with reduced frontal white matter volume (23), and it has been suggested that early pubertal timing creates a vulnerable state for the emergence of mental health difficulties (17). Similarly, accelerated pubertal tempo has been associated with greater cortical thinning (24), which in turn has been related to depression onset (25). According to this theoretical model, brain development would mediate the relationship between puberty and internalizing problems, with transactional (i.e., bidirectional) processes between brain development and internalizing problems (5). However, given the general scarcity of literature on the relationship between puberty, brain structure, and mental health, in the current article, we also review studies testing different models and consider all studies that have simultaneously investigated the relationship between puberty, brain structure, and mental health in adolescence.

More recently, research has also attempted to establish the utility of puberty as a predictor of adolescent mental health using a precision psychiatry approach. This approach moves away from the estimation of group-level effects and toward more complex prediction models that can yield individualized risk estimates so as to accurately discriminate between individuals with and without a target outcome (26). These models could also help determine the minimal set of features required to accurately make a prediction, which is needed to make cost-effective decisions in clinical practice (27).

Our review includes 2 parts. In the first part, we provide a systematic review of existing research on puberty, brain structure, and mental illness in young people. We included studies investigating 1) whether puberty and early psychopathology explain structural brain alterations, and 2) whether puberty and brain development produce a high-risk state for the emergence of mental illness in young people. In the second part, we review existing evidence on the value of puberty in models predicting individual-level mental health outcomes in adolescents, adopting a precision psychiatry approach and aiming to create a pooled estimate for the predictive value of puberty.

## Methods

We conducted 2 searches according to PRISMA (Preferred Reporting Items for Systematic Reviews and Meta-Analyses) reporting guidelines (28). The first is a systematic search of case-control, cohort, and cross-sectional studies that investigated puberty, brain structure, and mental health in young people with and without mental health difficulties. This search was preregistered on PROSPERO (29). The second was a search of risk calculation tools that used puberty in predictive models of mental health outcomes in young people. For both reviews, we searched PubMed, Web of Science, and PsycINFO for publications up until February 1, 2024. The first search included records from when first available, while the second search was limited from January 1, 2021, because it built on existing reviews of prediction models for mental health outcomes covering until the end of 2020 (30, 31, 32). Detailed methods for the 2 reviews, including search strings, gray literature search, and inclusion and exclusion criteria are presented in the Supplement. We assessed study quality using 1) the Newcastle–Ottawa scales for cross-sectional and cohort studies (33), and 2) the Prediction model Risk Of Bias ASsessment Tool (PROBAST) (34).

## Results

### Relationship Between Puberty, Brain Structure, and Psychopathology in Young People

**Figure 1:**
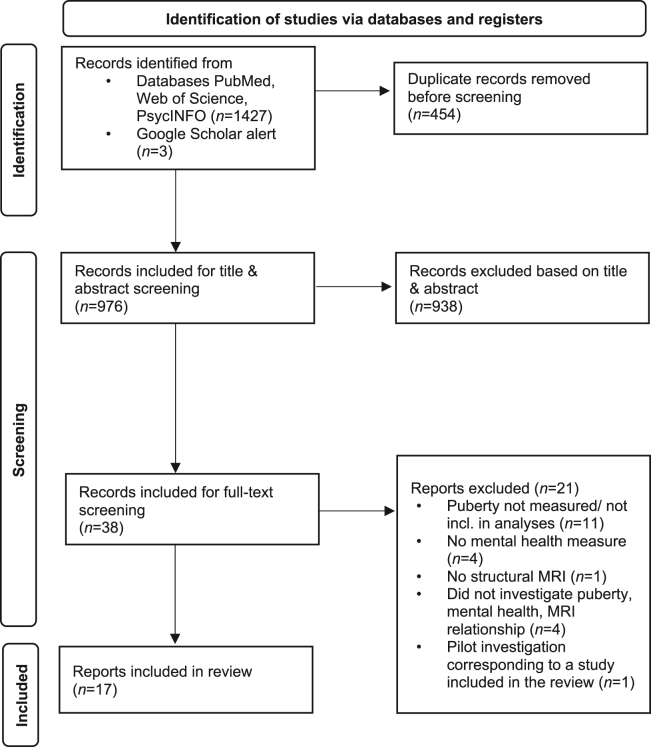
PRISMA (Preferred Reporting Items for Systematic Reviews and Meta-Analyses) flow diagram for the systematic review on puberty, brain structure, and psychopathology in young people. MRI, magnetic resonance imaging.

We identified 17 eligible studies (Figure 1), including 9 cross-sectional studies and 8 longitudinal cohort studies published between 2004 and 2023. All studies were rated “good quality,” as described in Tables S1 and S2. We grouped papers according to the outcome investigated. First, we describe studies that investigated whether/how puberty and mental illness explain brain structural changes (Table 1). Then we describe studies that investigated whether puberty and brain structure explain mental health outcomes (Table 2). One study (35) tested both types of association in their data (Table 3), which we describe at the end. Note that the search did not identify any study examining the 3-way relationship between pubertal tempo, brain structure, and mental health problems; all included studies investigated pubertal timing alone. A detailed description of the methods and findings of each study is presented in the Supplement.

**Table 1:** Studies Included in the Systematic Review Investigating Brain Structural Outcomes

Author, Year	Cohort/Study Population	Age Range, Years	Female,%	Ethnicity, %	Follow-Up Schedule	Puberty Measures	Mental Health Measures	MRI Measures	Software Used	Key Findings
Ahn *et al.*, 2007 (38)	Youth diagnosed with BPD (*n* = 46) and with no psychiatric diagnosis (*n* = 22)	6–16	BPD group: 43%, control group: 45%	BPD group: 98% White; control group: 91% White	NA	Tanner stage to divide sample into 2 groups: prepubertal (stage 1) and pubertal (stage 2–5)	K-SADS-E, self- and parent report; YMRS; GAF: past and current, self-report	Basal ganglia log volumes and asymmetry indices	Manual segmentation using Cardviews software (71)	Larger nucleus accumbens volume in prepubertal children diagnosed with BPD compared with control group; no differences for pubertal children.
Glenn *et al.*, 2022 (39)	Latina girls (*n* = 39) recruited from the Inland Empire region of Southern California	8–13	100%	100% Latina	NA	Tanner stage, self- and parent report	SCARED, self- and parent report	dMRI: FA in cingulum and uncinate fasciculus	FSL, DTIFIT	Parent- (but not self-) reported anxiety symptoms associated with lower cingulum FA, only significant in prepubertal girls.
Rogers *et al.*, 2019 (40)	Youth diagnosed with CD (*n* = 124) and with no psychiatric diagnosis (*n* = 174)	9–18	CD group: 48%; control group: 59%	Not reported, study sites in the United Kingdom, Germany, and Switzerland	NA	PDS, dichotomized to divide sample into 2 groups: pre/early and mid/late/post pubertal	K-SADS-PL, self- and parent report	dMRI: FA, MD, AD, RD	FSL diffusion toolkit	Youth with CD exhibited higher AD in corpus callosum and lower RD and MD in anterior thalamic radiation compared with the control group, independent of puberty.
Santos *et al.*, 2022 (41)	Adolescents with recent history of concussion (*n* = 55) (concussion 7.1 ± 2.1 days before scan) and age- and sex-matched adolescents with no current or history of concussion (*n* = 50)	12.5–17.9	Concussion group: 42%; control group: 44%	Concussion group: 87% Caucasian; control group: 73% Caucasian	NA	PDS, self-report	CDRS, self-report; SCARED, self-report	dMRI microstructure: NDI in emotion regulation tracts: left/right CB, *F*_min_, left/right uncinate fasciculus	NODDI toolbox (version 1.0.5)	Participants with concussion showed higher anxiety and lower NDI, with those at more advanced pubertal stages showing greater differences in the left and right frontal CB and left and right *F*_min._ compared with control participants. No effects for depressive symptoms.
Thomas and De Bellis, 2004 (36)	Children with history of maltreatment and PTSD (*n* = 61) and children with no history of trauma and no psychiatric diagnosis (*n* = 121) (2 age- and gender- matched control participants for each PTSD participant)	4.3–17	PTSD group: 51%; control group: 49%	PTSD group: 11% African American, 13% biracial, 75% White; control group: 9% African American, 15% biracial, 76% White	NA	Tanner stage	Detailed trauma interview; K-SADS-PL, self- and guardian-report	Pituitary volume	Manual segmentation using BrainImage	Pituitary volume was similar for prepubertal PTSD and control participants, whereas pubertal and postpubertal participants with PTSD showed significantly larger pituitary volume compared with control participants.
Weems, *et al.*, 2013 (37)	Youth diagnosed with PTSD (*n* = 24) and age- and gender-matched control participants with no psychiatric diagnosis (*n* = 24)	7–14	42%	PTSD at baseline: 25% African American, 4% Asian, 58% Euro-American, 12.5% Hispanic. PTSD at follow-up: 40% African American, 7% Asian, 47% Euro-American, 7% Hispanic	1 follow-up after 12–18 mo; *n* = 15 participants with PTSD	Tanner stage at baseline, self-report	PTSD Reaction Index to screen PTSD group for inclusion, CAPS-CA, self-report	Amygdala volumes	Manual tracing using BrainImage	Nonsignificant baseline group differences in amygdala volume. Significant quadratic (and to a lesser extent linear) association between Tanner stage and amygdala volume (prepubertal smaller amygdala, increased volume at stages 2 and 3, decreased volume at stage 4) in the PTSD group; no significant association for the control group. For children with PTSD symptoms, pubertally less mature youths (Tanner stages 1 and 2) showed volume increases, while more mature youth (stages 3 and 4) showed volume decreases over time.

AD, axial diffusivity; BPD, bipolar disorder; CAPS-CA, Clinician-Administered PTSD Scale for Children and Adolescents; CB, cingulum bundle; CD, conduct disorder; CDRS, Children’s Depression Rating Scale; dMRI, diffusion MRI; FA, fractional anisotropy; *F*_min_, forceps minor; GAF, Global Assessment of Functioning; K-SADS-E, Kiddie Schedule for Affective Disorders and Schizophrenia, Epidemiological version; K-SADS-PL, K-SADS Present and Lifetime version; MD, mean diffusivity; MRI, magnetic resonance imaging; NDI, neurite density index; PDS, Pubertal Development Scale; PTSD, posttraumatic stress disorder; RD, radial diffusivity; SCARED, Screen for Child Anxiety Related Disorders; YMRS, Young Mania Rating Scale.

**Table 2:** Studies Included in the Systematic Review Investigating Mental Health Outcomes

Author, Year	Cohort/Study Population	Age Range, Years	Female, %	Ethnicity, %	Follow-Up Schedule	Puberty Measures	Mental Health Measures	MRI Measures	Software Used	Key Findings
Dehestani *et al.*, 2023 (18)	ABCD Study (*n* = 10,167)	9–13	48%	Not reported^a^	NA	Baseline PDS, parent report; salivary/testosterone, DHEA: predicting group-level age based on physical/hormonal puberty features and subtracting each participant’s chronological age to reflect pubertal timing (“puberty age gap”)	CBCL, parent report	Baseline cortical and subcortical volumes, CT, and surface area: predicting group-level age based on brain features and subtracting each participant’s chronological age to reflect relative brain maturation (“brain age gap”)	FreeSurfer 5.3.0	Early pubertal timing associated with accelerated brain maturation (especially, subcortical and frontal regions in females and subcortical regions in males) and with more mental health issues in both sexes. Brain age not significantly associated with mental health problems and not a significant mediator in the relationship between pubertal timing and mental health problems.
Ellis *et al.*, 2019 (44)	iCATS study; subsample of children with androgen levels in upper or lower tertials (*n* = 88)	8.3–9.7	52%	Not reported^b^	NA	SMS-PR based on Tanner stage; salivary DHEA and testosterone	CDI-2, self-report	Hippocampal and whole-brain volumes	FreeSurfer 5.3	Larger right hippocampal volume mediated the relationship between early pubertal timing (relatively high testosterone level at young age) and increased depressive symptoms, for girls only.
MacSweeney *et al.*, 2023 (48)	ABCD Study, participants with available quality-controlled baseline puberty data and follow-up neuroimaging data (*n* = 5727)	10–11	48%	2.5% Asian, 10.2% Black, 11.4% Mixed, 0.7% Native Hawaiian/Native Pacific Islander/American Indian/Alaskan Native, 3.8% Other, 71.4% White	2 follow-ups at 1-year interval; exact *N* not reported, *N* = ∼5000 at each time point	Baseline PDS, parent report: regressed PDS total score on age for girls and boys separately and took standardized residuals as continuous measure of pubertal timing	CBCL, parent report	Cortical and subcortical volumes; dMRI measures: FA, MD at follow-up 1	FreeSurfer; Atlastrack	Early pubertal timing associated with higher depressive symptoms 2 years later, stronger in females (effect remained significant after controlling for BMI, parental depression, and family income in females, not in males). Structural brain measures did not mediate the association between early pubertal timing and depressive symptoms.
Murray *et al.*, 2016 (17)	iCATS study; subsample of children with androgen levels in upper or lower tertials (*n* = 95)	Not reported, mean 9.5 ± 0.34 SD	53%	Not reported^b^	NA	Sexual maturity status, parent report (SMS-PR); salivary testosterone, DHEA, DHEA-S	SCAS, self-report	Pituitary volume	Manual segmentation using ANALYZE (Mayo Clinic)	Larger pituitary volume mediated the relationship between early pubertal timing (relatively high DHEA and DHEA-S levels at young age) and increased social anxiety. Controlling for Tanner stage did not change conclusions. No sex effects.
Nguyen *et al.*, 2016 (45)	NIH MRI Study of Normal Brain Development, “healthy developing children” across United States, with mental health data available (*n* = 207)	6.1–19.7	67%	Not reported^c^	2 follow-ups at 2-year intervals; *n*s for individual time points not reported, *n* = 78 participants with any follow-up time point	PDS, physician-rated; salivary testosterone, estradiol	CBCL, parent report; YASR, self-report	Structural covariance between whole-brain cortical thickness and amygdala volume	CIVET pipeline; manual segmentation (ANIMAL procedure)	No effects of pubertal stage, age, sex, estradiol levels. Negative amygdala -medial prefrontal cortex covariance mediated the relationship between higher testosterone and more symptoms of aggression, no effects on anxiety and depression symptoms.
Nguyen *et al.*, 2019 (46)	NIH MRI Study of Normal Brain Development, ”healthy developing children” across United States (*n* = 152)	6.09–18.13	38%	Not reported^c^	2 follow-ups at 2-year intervals; *n* = 64 at follow-up 1, *n* = 58 at follow-up 2	PDS, physician rated; salivary testosterone, estradiol, DHEA	CBCL, parent report	Structural covariance between whole-brain cortical thickness and amygdala volume	CIVET pipeline (v 1.1.9); manual segmentation (ANIMAL procedure)	Estradiol-related cortico-amygdala covariance (tended to switch from positive to negative covariance with rising estradiol) was not related to anxiety or depression symptoms; no effects of pubertal stage or sex.
Okada *et al.*, 2020 (42)	Tokyo TEEN cohort (population-based birth cohort study), representative subsample (*n* = 203)	Not reported, mean 10.2 ± 0.3 SD	48%	98.3% mother’s country of origin Japan; 93.9% father’s country of origin Japan (72)	1 follow-up after 2 years; *n* = 203	Tanner stage, parent report (used pubic hair score as outcome)	SDQ, parent report	Baseline gray matter volume, whole-brain analysis	SPM12	Smaller subgenual anterior cingulate cortex volume mediated the relationship between earlier pubertal timing and elevated psychological difficulties at follow-up in females, not in males; no effects for baseline psychological difficulties.
Whittle *et al.*, 2012 (9)	Children with no DSM-IV diagnosis of depression (*n* = 155), recruited from schools across metropolitan area in Melbourne, Australia	Not reported, mean 12.7 ± 0.5 SD	46%	Not reported	1 follow-up after 2.6 ± 0.3 years; *n* = 141	Baseline: PDS, parent report. Follow-up: PDS parent and self-report; schematic drawings based on Tanner stage, self-report	CES-D, self-report	Baseline pituitary volumes	ANALYZE (Mayo Clinic)	At baseline, early pubertal timing associated with larger pituitary gland volume and more depressive symptoms, with stronger effect in girls and no mediation effect. Larger pituitary gland volume at baseline mediated the relationship between early pubertal timing and higher depressive symptoms over time in both sexes.
Wiglesworth *et al.*, 2023 (47)	ABCD Study (*n* = 9985)	8.9–11	8%	Baseline: 6.5% Asian, 20% Black/African American, 18.5% Hispanic/Latinx, 3.5% Native American, 6.2% Other, 0.6% Pacific Islander, 77.4% White. Follow-up: 6.2% Asian, 18.5% Black/African American, 17.7% Hispanic/Latinx, 3.9% Native American, 0.8% Pacific Islander, 17.7% Hispanic/Latinx, 78.8% White	1 follow-up after 2 years; *n* = 4800	PDS, parent report	CBCL, parent report; K-SADS-COMP, self- and parent report	CT development of 34 ROIs, averaged across hemispheres	FreeSurfer	No significant 3-way interaction between sex, CT change, and pubertal development in predicting internalizing symptoms or suicide ideation at follow-up. CT development not associated with mental health measures. More advanced pubertal stage at baseline predicted increased internalizing symptoms, but not suicide ideation at follow-up.
Zipursky *et al.*, 2011 (43)	Children with no DSM-IV depression diagnosis (*n* = 155), recruited from schools across metropolitan Melbourne, Australia, same cohort as Whittle *et al.* (9)	Not reported, mean 12.7 ± 0.5 SD	46%	Not reported	1 follow-up after 2.6 ± 0.3 years; *n* = 141	PDS, parent report	K-SADS-COMP; CES-D; BAI; CBCL, self-report	Baseline pituitary volume	ANALYZE (Mayo Clinic)	Larger pituitary volumes predicted more anxiety symptoms over time. Pubertal stage was associated with pituitary volume, but not significant in predicting anxiety symptoms beyond pituitary volume.

ABCD, Adolescent Brain Cognitive Development; ANIMAL, automated nonlinear image matching and anatomical labeling; BAI, Beck Anxiety Inventory; BMI, body mass index; CBCL, Child Behavior Checklist; CDI-2, Child Depression Inventory 2; CES-D, Center for Epidemiological Studies Depression Scale; CT, cortical thickness; DHEA, dehydroepiandrosterone; DHEA-S, DHEA-sulfate; dMRI, diffusion MRI; FA, fractional anisotropy; iCATS, Imaging Brain Development in the Childhood to Adolescence Transition Study; K-SADS, Kiddie Schedule for Affective Disorders and Schizophrenia; K-SADS-COMP, K-SADS-Computerized version; MD, mean diffusivity; NIH, National Institutes of Health; PDS, Pubertal Development Scale; ROI, region of interest; SCAS, Spence Children’s Anxiety Scale; SDQ, Strength and Difficulties Questionnaire; SMS-PR, Sexual Maturity Status-Parent Report; YASR, Young Adult Self-Report.

a The baseline ABCD sample has the following ethnicity distribution: 5.3% Asian; 16.6% Black; 23.2% Hispanic; 5.4% Native Hawaiian/Native Pacific Islander/American Indian/Alaskan Native; 49.5% White (73).

b The full Imaging brain development in the iCATS sample consists of predominantly White participants (74).

c The NIH MRI Study of normal brain development baseline sample has the following ethnicity distribution: 2% Asian, 11% Black, 12% Hispanic, 1% Native Hawaiian/Native Pacific Islander/American Indian/Alaskan Native, 72% White (75).

**Table 3:** Studies Included in the Systematic Review Investigating Brain Structural and Mental Health Outcomes

Author, Year	Cohort/Study Population	Age Range, Years	Female, %	Ethnicity, %	Follow-Up Schedule	Puberty Measures	Mental Health Measures	MRI Measures	Software Used	Key Findings
Picci *et al.*, 2023 (35)	Healthy children and adolescents (*n* = 137), subsample of participants who completed an MRI scan as part of the Developmental Chronnecto-Genomics study	9–17	49%	1% Asian, 4% Black, 4% Indigenous, 4% multiracial, 85% White, 2% not reported	NA	Salivary DHEA	TSCC, self-report	Anterior and posterior pituitary volume	FreeSurfer to extract total intracranial volume, manual tracing of pituitary	Model 1: lower levels of salivary DHEA mediated the association between higher trauma-related anxiety and smaller anterior pituitary volume; more anxiety symptoms related to larger posterior pituitary volume. Model 2: no direct or indirect effects of pituitary volumes or DHEA on anxiety, depression, or posttraumatic stress symptoms.

DHEA, dehydroepiandrosterone; MRI, magnetic resonance imaging; NA, not applicable; TSCC, Trauma Symptom Checklist for Children.

#### Do Puberty and Early Psychopathology Explain Brain Structural Alterations?

Studies focusing on brain structure as an outcome (*n* = 6) could be grouped into studies investigating volumetry of a single structure that is thought to be related to puberty [pituitary (36), amygdala (37), basal ganglia (38)] and studies investigating white matter integrity through diffusion tensor imaging (39, 40, 41). The latter were diverse in terms of mental health and were often whole-brain analyses. The search did not identify any study examining the 3-way relationship between pubertal tempo, brain structure, and mental health problems, and all included studies investigated pubertal timing alone.

The volumetry studies were consistent in identifying interactions between puberty status and mental health in their effects on structure volume, but the form of this interaction differed. For example, Ahn *et al.* (38) showed that nucleus accumbens volumetric differences between participants with bipolar disorder (BPD) and control participants were only significant for prepubertal children, while Thomas and De Bellis (36) showed that pituitary volume increases compared with control participants were only present for postpubertal participants with posttraumatic stress disorder (PTSD). Findings from the 3 studies investigating white matter integrity were inconsistent, with effects of mental illness on white matter being independent of pubertal stage (40), more pronounced in prepubertal participants (39), or more pronounced at more advanced pubertal stages (41).

#### How Do Puberty and Brain Development Explain the Emergence of Mental Illness in Young People?

Ten studies considered mental illness measures as the outcome. Of these, 2 considered interaction models and tested whether the association of structural brain measures with mental health differed according to pubertal status, and 8 investigated whether brain structure mediated the relationship between pubertal stage and mental health. The studies investigated different brain structure measures, making it difficult to synthesize the evidence on neuroimaging findings. Five studies exclusively focused on gray matter volumes; of these, only 1 included a whole-brain analysis (42) while 4 focused on a priori-defined regions [pituitary (9,17,43) and hippocampus (44)]. Two analyses included structural covariance between whole-brain cortical thickness and amygdala volume (45,46), 1 study investigated cortical thickness in 34 regions (47), and 2 studies investigated multiple morphometric parameters [gray matter volumes, cortical thickness, cortical surface area (18); gray matter volumes, measures (48)]. We grouped studies according to the pubertal parameters that were investigated: pubertal hormones, observable physical development, or both.

Two studies found a link between early exposure to pubertal hormones and internalizing symptoms, mediated by structures sensitive to these hormones, such as the pituitary (17) and the hippocampus (44). Five studies used measures of observable pubertal development with parent- and child-report questionnaires, and 2 of these reported mediating effects of pituitary volume for both sexes (9) and subgenual anterior cingulate cortex volume for females (42) in the relationship between early puberty and mental illness. However, there was no evidence for a moderating effect of pituitary volume in this relationship (43). Two analyses of the Adolescent Brain Cognitive Development (ABCD) Study cohort also reported an association between early puberty and internalizing symptoms, but no mediating (48) or moderating (47) effects of structural brain measures. Finally, from the 3 studies that investigated both hormonal and physically observable puberty features, 2 investigations of 1 cohort found no significant effects of puberty measures or cortico-amygdala covariance on internalizing symptoms (45,46), while another study reported a link between early pubertal timing and more mental health problems, but not a mediating effect of brain structure (18).

Lastly, Picci *et al.* (35) assessed puberty with salivary DHEA and tested 2 models with different outcomes: 1) pituitary volume, and 2) anxiety, depressive, or PTSD symptoms. First, lower levels of salivary DHEA mediated the association between higher trauma-related anxiety and smaller anterior pituitary volume, whereas higher anxiety symptoms were related to larger posterior pituitary volume. Second, higher DHEA was related to larger anterior pituitary volume, while there were no direct effects of DHEA on either symptom domain and no mediation by pituitary volumes (26).

Taken together, while studies investigating mental health as the outcome do confirm that early puberty is associated with mental health problems, they provided inconsistent evidence about the possible mediating role of brain structural measures and suggested that this may differ between the sexes, with no evidence for a moderating role of brain structure measures. Overall, the findings seem consistent in highlighting the importance of considering pubertal stage when evaluating vulnerability for mental disorders and brain development, but point to the need for larger, more standardized evaluations of puberty.

### Risk Prediction Models Including Puberty as an Independent Predictor of Adolescent Mental Health

**Figure 2:**
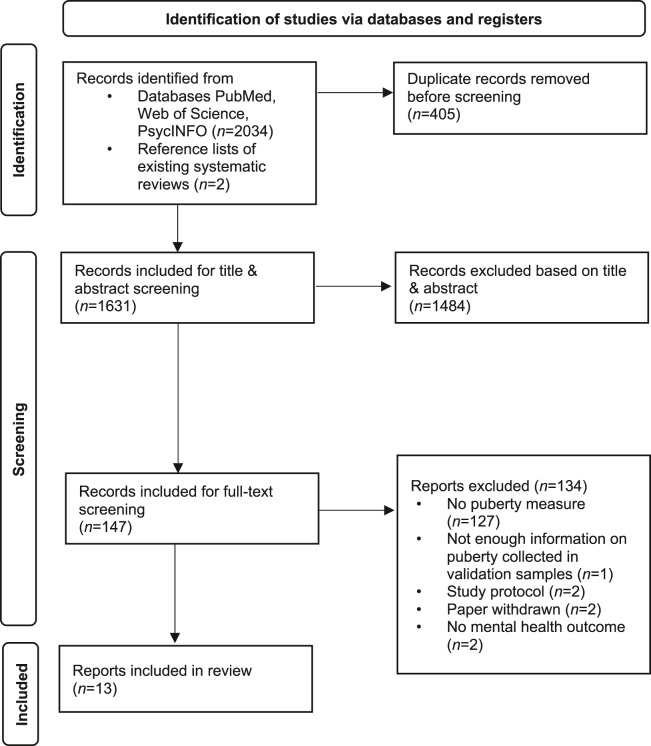
PRISMA (Preferred Reporting Items for Systematic Reviews and Meta-Analyses) flow diagram for the review of risk prediction modeling studies.

We identified 13 studies published between 2018 and 2024 that used pubertal measures, including 1 study published before 2021 that we identified in the gray literature search (Figure 2). Twelve studies developed new risk calculation tools, and 1 study validated an existing tool to predict adolescent mental health (Table 4). Risk of bias ranged from low (*n* = 7) to unclear (*n* = 6), as described in Table S3. All studies included pubertal stage relative to age in their model (i.e., pubertal timing), but no study used pubertal tempo characteristics to predict adolescent mental health outcomes. Pooled estimates of the predictive utility of puberty were not obtainable because the necessary quantities were commonly not reported (49). Accordingly, we provide a narrative synthesis of evidence on the predictive value of puberty when included in these prediction models for mental health outcomes and present a detailed description of these results in the Supplement. First, we consider models where physical staging or staging and biomarkers were jointly considered followed by models that only included biomarkers of puberty.

**Table 4:** Selected Prediction Modeling Studies

Author, Year	Prediction Model Outcome	Number of Outcome Categories	Sample	Age Range, Years	Predictor Type	Puberty Measures	Model Type	Validation	Model Classification Performance^a^	Predictive Performance of Puberty
de Lacy *et al.*, 2023 (56)	Cases of either anxiety, depression, disruptive behaviors, PTSD, or attention-deficit/hyperactivity disorder diagnosed from K-SADS	2	HBN study assessing young people with at least 1 behavioral concern (*n* = 1120)	5–21	160 predictors including neural, prenatal, developmental, physiological, sociocultural, environmental, emotional, and cognitive features	Interview-based PDS, ages 6–11 parent report, age >11 self-report	Machine learning: deep learning, GBT-based learning, logistic regression	Dataset divided into development (*n* = 783) and validation (*n* = 336) samples	AUROC ≥ 0.94 for the best performing model for each disorder in validation sample	Puberty not among the top 1 to 6 predictors
Hawes *et al.*, 2022 (52)	Depression or anxiety at age 15 based on DSM-IV and DSM-5 diagnoses from K-SADS	2	Stony Brook temperament study (*n* = 374)	3–15	Psychopathology, temperament and personality, life stress, family and interpersonal, EEG, cortisol, parental history of psychiatric diagnosis and personality	DHEA and testosterone (ages 9 and 12); estradiol (age 12); PDS, mother-report (ages 9 and 12); Tanner stage, self-report by drawings (age 12)	L2 (Ridge) penalized logistic regression for classification, with CCA components as inputs	10-fold cross-validation	Varied depending on which time point(s) were used to predict outcome, AUROC = 0.599–0.751 for depression, AUROC = 0.621–0.812 for anxiety	CCA: PDS assessed at ages 9 and 12 among top predictors, included as a feature in the model
Ho *et al.*, 2022 (53)	Presence of depressive symptoms at baseline and after 1 year, based on CBCL depressive problems subscale standardized scores	2	ABCD cohort (*n* = 7995)	8–11	rsfMRI, fMRI connectivity, demographic, family history, sleep, substance use	PDS	Machine learning: linear (EN) and nonlinear (GBT) predictive models	Dataset divided into development (75%) and validation (25%) samples	Minimizing MAE, baseline depression MAE = 3.757 (for EN), 3.761 (for GBT); depression at 1-year follow-up MAE = 4.255 (EN), 4.262 (GBT)	Included as feature in the model, no more details
Mongan *et al.*, 2021 (62)	Transition to psychotic disorder at/before age 18 in high-risk adolescents, diagnosis of psychotic disorder assessed with CAARMS interview	2	Clinical high-risk state (*n* = 334) and control (*n* = 14,901) groups from EU-GEI and ALSPAC	12–18	Proteomic and clinical	SHBG from blood samples	Machine learning; SVM model	Repeated nested cross-validation; dataset divided into development and validation samples (high-risk group, *n* = 49; control group, *n* = 86)	AUROC = 0.95; model based on the 10 most predictive proteins for train AUROC = 0.99; for test AUROC = 0.92	SHBG not significant as predictor
Mürner-Lavanchy *et al.*, 2024 (61)	Whether a patient had engaged in nonsuicidal self-injury at least 5 times in the past year (yes/no), assessed with the German version of the SITBI-G	2	Adolescents with nonsuicidal self-injury (*n* = 149) and control participants (*n* = 40), recruited from a cohort study with adolescents seeking help at an outpatient clinic for risk-taking and self-harming behavior	12–17	Oxytocin, DHEA, β-endorphin, free triiodothyronine, leukocytes, heart rate variability, pain sensitivity	DHEA from blood samples	Machine learning: logistic regression, elastic net regression, random forests, GBT	5-fold cross-validation	AUROC = 0.67–0.7 for different model types	DHEA not among top 3 predictors
Nichols *et al.*, 2018 (58)	A diagnosis of depression in clinical records	2	Matched case-control sample from UK THIN database (67,321 cases; 192,135 controls)	15–24	42 predictors from electronic primary care records including psychiatric symptoms, comorbid disorders, social factors, substance use	Early/late puberty present if a clinical code was ever recorded	Stepwise logistic regression	Dataset divided into development and validation samples (*n* = 31,241 cases, *n* = 89,113 controls)	AUROC similar for the 4 models; males ages 15–18 = 0.71 (95% CI 0.70–0.73), males ages 19–24 = 0.72 (95% CI 0.71–0.72), females ages 15–18 = 0.72 (95% CI 0.71–0.73), females ages 19–24 = 0.70 (95% CI 0.69–0.70)	Early/late puberty code not selected for final model
Rothenberg *et al.*, 2023 (50)	Internalizing or externalizing symptoms at ages 13 and 17 (CBCL, median split)	2	Parenting Across Cultures Project (includes 12 cultural groups from 9 nations); (*n* = 1176 families)	10–17	79 predictors based on bioecological model (mental health, micro- and macro-environment) assessed at age 10	PDS (age 10), self-report	Machine learning; SVM and random forest	Dataset divided into development and validation samples (*n* = 105–135)	MCCs, both algorithms yielded overfitting, SVM superior: for internalizing age 13 train = 0.76, test = 0.44; age 17 train = 0.65, test = 0.23; for externalizing age 13 train = 0.73, test = 0.46; age 17 train = 0.8, test = 0.44	PDS among top 15 most important predictors of internalizing symptoms at age 13
Su *et al.*, 2023 (51)	Self-harm (question derived from the ALSPAC) and suicide attempt (derived from the National Survey of Mental Health & Wellbeing) at ages 16 to 17	2	LSAC (*n* = 2890)	14–17	497 predictors including physical and mental health, puberty, sociodemographic factors, parenting, bullying, and social and school-related factors	Puberty scale, no further information, (ages 14–15), self-report	Machine learning: random forest	Dataset divided into development (70%) and validation (30%) samples	AUROC = 0.74 for self-harm, AUROC = 0.72 for suicide attempt in validation dataset	Puberty fifth most important feature for predicting self-harm, not among top 20 predictors for predicting suicide attempt
Toenders *et al.*, 2022 (54)	Depression onset at ages 16 and 19: sub- and full-threshold major depressive disorder based on Development and Well- Being Assessment	2	IMAGEN cohort (*n* = 2223; 180 participants with depression, 229 control participants)	14–19	Clinical, cognitive, environmental, structural MRI	PDS (age 14)	Penalized logistic regression (different levels of penalization)	Dataset divided into development and validation samples (*n* = 68 depression, 69 control)	AUROC = 0.70–0.72 (SD = 0.07–0.1) for different alpha levels in development dataset; AUROC = 0.68–0.72 for validation dataset	PDS listed as developmental predictor, no further information
Van Meter *et al.*, 2021 (59)	Diagnosis of bipolar disorder at 2-year follow-up (K-SADS)	2	High-risk youth (*n* = 473) from LAMS cohort	6–12	Demographic and clinical interview	PDS	Cox proportional hazards regression	Validation study, original model developed on independent cohort	AUROC = 0.67 (95% CI 0.61–0.72) for original model on new sample	PDS was not a significant predictor
van Velzen *et al.*, 2022 (55)	Suicidal thoughts and behaviors, with K-SADS parent-and child-reported suicidal thoughts and behaviors	2	ABCD cohort (*n* = 5885)	9–11	Sociodemographic, physical health, social, environmental, clinical psychiatric, cognitive, genetic (polygenic risk scores from saliva samples), task-based fMRI	PDS, self-report	Penalized logistic regression analysis	Dataset divided into development (2/3) and validation (1/3) samples	Children without a diagnosis and with suicidal thoughts and behaviors vs. children without (AUROC = 0.80–0.81), children with a psychiatric diagnosis vs. children without diagnosis (AUROC = 0.71–0.77). Models did not distinguish children with suicidal ideation from those with suicide attempts (AUROC = 0.49–0.58); for validation set, AUROC = 0.5–0.77.	PDS listed under selected features for child- and parent-report models
Wu *et al.*, 2022 (60)	ICD-10 diagnosis of bipolar disorder or ICD-10 diagnosis of depressive disorder	2	Patients first-time admitted to the SMHC (*n* = 261; 106 participants with depression, 101 participants with bipolar disorder)	10–18	Basic demographic data and peripheral markers in blood taken when first admitted	Estradiol, testosterone, LH, FSH	Binary logistic regression	Independent external validation with 255 matched patients from another hospital in China	AUROC = 0.785 for all adolescents; 0.816 for males; 0.793 for females; AUROC = 0.714 for validation set	Pubertal hormones not in final model, not significant
Xiang *et al.*, 2022 (57)	Depressive symptom trajectory groups over 2 years: “increasing,” “decreasing,” “persistently low,” and “persistently high,” based on CBCL	4	ABCD cohort (*n* = 4962)	9–12	Demographic, physical health, academic, environmental, social, traumatic and stressful events, perinatal factors, rsfMRI	PDS (ages 9–10)	Machine learning: GBM, extreme gradient boosting, random forest, k-nearest neighbor classifier	Dataset divided into development (80%) and validation (20%) samples	GBM model superior, multiclass macro-average AUROC = 0.77; multiclass micro-average AUROC = 0.90	PDS not among most important features in distinguishing trajectories of depression

ABCD, Adolescent Brain Cognitive Development; ALSPAC, Avon Longitudinal Study of Parents and Children; AUROC, area under the receiver operating characteristic curve; CAARMS, Comprehensive Assessment of At-Risk Mental States; CBCL, Child Behavior Checklist; CCA, canonical correlation analysis; DHEA, dehydroepiandrosterone; EEG, electroencephalography; EN, elastic net; EU-GEI, European Network of National Schizophrenia Networks Studying Gene-Environment Interactions; FSH, follicle-stimulating hormone; GBM, gradient-boosting machine; GBT, gradient-boosted trees; HBN, Healthy Brain Network; K-SADS, Kiddie Schedule for Affective Disorders and Schizophrenia; LAMS, Longitudinal Assessment of Manic Symptoms; LH, luteinizing hormone; LSAC, Longitudinal Study of Australian Children; MAE, mean absolute error; MCC, Matthew correlation coefficients; MRI, magnetic resonance imaging; PDS, Pubertal Development Scale; PTSD, posttraumatic stress disorder; rsfMRI, resting-state functional MRI; SHBG, sex hormone binding globulin; SITBI-G, Self-Injurious Thoughts and Behaviors Interview-German Version; SMHC, Shanghai Mental Health Center; SVM, support vector machine; THIN, The Health Improvement Network.

a 95% CI or SD are provided when reported.

Two studies provided information about the relative importance of puberty in prediction models. Variability in self-reported pubertal development was among the top 15 most important predictors for internalizing symptoms in early adolescence (50) and the fifth most important predictor for self-harm in later adolescence (51), but it was less relevant for later onset of internalizing symptoms (50) and for predicting suicide attempts (51). Four studies listed puberty as a predictor in their final models but did not provide information about the relative importance of puberty as a factor. Pubertal timing assessed with the Pubertal Development Scale in early adolescence, but not with hormonal measures, was predictive of depression and anxiety symptoms later in adolescence (52). Variations in puberty measured with the Pubertal Development Scale were also predictive of a diagnosis of depression in children (ages 8–11 years) at the first assessment and predictive of its prognosis after 1 year (53) and of the onset of depressive symptoms at ages 16 and 19 years (54). Puberty was utilized in prediction models that distinguished children with suicidal thoughts and behaviors from children without these characteristics and children with a psychiatric diagnosis from children without a diagnosis, although the models did not discriminate children with suicidal ideation from those with suicide attempts (55).

Four studies that investigated physically observable pubertal features did not eventually select them as features for inclusion in the final prediction model. Puberty was not among the first top 6 predictors for different psychiatric diagnoses (56) in 1 study, and it was of negligible relevance to classification of children into depressive symptom trajectory groups (57), prediction of a diagnosis of depression based on primary care records (58), or prediction of BPD emergence over 2 years (59). Lastly, 3 studies assessed puberty with hormonal or proteomic features and did not find them to be significant in the final model for the prediction of a diagnosis of BPD (60), nonsuicidal self-injury (61), or transition to psychosis (62).

In conclusion, of the 13 prediction modeling studies reviewed, 7 did not find variations in pubertal timing to be relevant in the prediction of adolescent mental health outcomes. This may partially be due to how individual studies conceptualized and measured puberty, with hormonal and proteomic pubertal features being less predictive than physically visible development. In contrast, 6 of 10 prediction modeling studies that assessed pubertal timing by means of physically observable characteristics did select puberty for the final prediction model, pointing to its potential relevance for the prediction of psychiatric diagnoses, depression, anxiety, and suicidal ideation in adolescence.

## Discussion

To our knowledge, this is the first systematic review on the relationship between puberty, brain structure, and mental health difficulties in adolescence and the first to also review prediction modeling studies of the role of puberty in the individualized prediction of adolescent mental health. Our first finding is that while both pubertal timing and mental health problems are associated with structural brain alterations, whether and how pubertal and psychopathological processes interact to explain these alterations remains to be established. Similarly, we found that studies were also inconsistent as to whether structural brain measures mediated the relationship between early puberty and mental health problems in adolescence. Our second main finding is that puberty measured by means of physically observable characteristics rather than by hormonal measures may be an important feature in the individualized prediction of depression, anxiety, and suicidal ideation and behavior in adolescents.

Both pubertal timing and mental health symptoms have been found to correlate with brain structural measures, but evidence on whether and how pubertal and psychopathological processes interact to explain differences in subcortical volumes (35, 36, 37, 38) and white matter structure (39, 40, 41) remains inconsistent. An important limitation to consider when evaluating this evidence is that, with the exception of 1 study (37), all studies investigating these effects were cross-sectional. Existing literature on structural brain alterations in adolescents with mental health problems is similarly inconsistent and points to the presence of different developmental trajectories throughout puberty across individuals and symptom domains (6,14,15), which cannot be captured cross-sectionally. In addition, a number of limitations are implicit in assessing puberty only cross-sectionally given the potential for erroneous ratings and the possibility of missing regressing development, i.e., receding developmental status between different time points (63), which can be captured by longitudinal investigations. Therefore, there is a real need for longitudinal studies that investigate whether pubertal processes normalize or intensify maladaptive neural growth and when differences in brain structure emerge.

Our review found evidence that early-maturing physical characteristics increase the risk for mental health difficulties in adolescence, with some evidence that structural brain parameters mediate, but do not moderate, this relationship (9,17,42, 43, 44,47). An enlarged pituitary volume was the brain measure most consistently reported (in 3 samples) in relation to PTSD diagnosis mid- and postpuberty (36), and it was found to mediate the relationship between early puberty and anxiety (17) and depressive symptoms (9). This is consistent with evidence that early maturation of the HPG and HPA axes, of which the pituitary is an integral part, represents a risk factor for adolescent mental disorders (6,17). Moreover, the HPG and HPA axes interact to mediate secretion of the stress hormone cortisol (7), and an enlarged pituitary volume has been associated with dysfunctional stress-related reactivity of the HPA axis (64). Research shows that stress-related HPA axis dysfunction is associated with depression (65) and that individuals exposed to early adversity and trauma show altered HPA axis function (66), suggesting a mechanism by which the pituitary may act as a mediator in the association observed between early puberty and poor mental health. One study found that lower DHEA levels mediated the association between more anxiety symptoms and smaller anterior pituitary volume (35), and relatively low DHEA levels throughout development may increase vulnerability to stress-related psychopathology (35,56). It is important to note that the informative value derived from the pituitary volume as the most consistently reported brain measure is limited because studies investigated the pituitary a priori based on its role in regulating puberty instead of identifying its importance relative to other structures in whole-brain analyses. Therefore, future studies with whole-brain analyses are needed to validate the importance of brain structures such as the pituitary relative to other regions in the relationship between puberty and mental health. Of note, the literature included is not informative regarding the role of other endocrine systems that undergo changes during puberty and are relevant for mental health, such as the thyroid axis (8), highlighting the need for future investigation.

Findings from the ABCD Study cohort provide further support for a link between early-maturing physical characteristics and internalizing symptoms in adolescence (47,48), with 1 investigation also including pubertal hormones and externalizing symptoms (18). However, none of the studies on the ABCD cohort found any mediating or moderating effects of structural brain measures. This may suggest that structural brain parameters do not play a role in increasing risk for mental health difficulties. On the other hand, it is also possible that large interindividual variability in puberty-related neurodevelopment (67) produces inconsistent and null effects at a group level. Some brain features may be relevant for some but not all young people or only to specific developmental periods, suggesting that moving from group-level investigations to a more individual-centered approach could be beneficial (48). Several cortical and subcortical features that are reorganized during puberty have been found to be altered in mental illness (14), yielding a wide variety of potentially relevant features. A lack of consistency can be expected given that studies commonly focus on only one or a few neuroimaging parameters. In addition, our search was limited to the investigation of brain structure, whereas functional and organizational brain changes occur during puberty and are also likely to be relevant (11), and studies investigating the link between puberty and brain development or brain development and mental health could also enrich knowledge on this topic. However, these were outside the scope of the current review given our original aims. Future studies may benefit from exploring multiple brain measures in prediction modeling and potentially identify neuroimaging-grounded subtypes that can better capture individual differences (26).

One cohort study assessed puberty with both hormonal and physically observable features, and pubertal timing and brain structure (45,46) did not predict internalizing symptoms, possibly due to the low variance in internalizing symptoms across participants (46). All other studies identified early puberty as a risk factor for adolescent mental health problems. It is interesting that previous research has reported particularly negative effects of early puberty for girls (5,21,68), and a recent proof-of-principle paper reported main effects of puberty and psychopathology on a latent whole-brain structural magnetic resonance imaging measure in females only (69). Some studies included in this review found stronger effects for girls (9,42,44,48), who may be particularly exposed to psychosocial risks such as bullying and unwanted attention as physical changes start to occur when they enter puberty (5). However, most studies found effects of atypical pubertal timing on mental health regardless of sex, and smaller effects reported by some for boys could reflect their prepubertal state at the time of assessment (48). Interestingly, any negative effect of early pubertal timing may evolve over time, with some studies finding stronger prospective than concurrent associations with mental health outcomes (9,37,42,70), again highlighting the importance of longitudinal investigations. Here, we have provided a synthesis of the existing evidence regarding the 3-way relationship between puberty, brain development, and mental health and showed that not many studies included measures of all three of them. Therefore, the evidence reviewed is not sufficient to establish with certainty whether brain structure acts as a mediator of the relationship between early pubertal timing and mental health difficulties in adolescence, as posed by the conceptual model of Pfeifer and Allen (5). Furthermore, the longitudinal studies included found only limited evidence that pubertal and brain developmental processes transactionally increase the risk of internalizing problems in adolescence. These gaps highlight the need for more research on the role of a key biological event such as puberty given the high risk that adolescence (and possibly pubertal and associated neurodevelopmental changes) poses for the emergence of mental health problems (3,5).

To explore the predictive utility of puberty, we reviewed prediction modeling studies that included pubertal features in the individualized prediction of mental health problems in young people. Puberty was found to be one of the most significant predictors of internalizing symptoms (50,52), depressive symptoms (53,54), and suicidal thoughts and behaviors (55). Puberty was relevant to the prediction of psychiatric diagnoses in one (55) but not in another investigation (56), and it was among the most important predictors of self-harm when measured by means of self-report (51) but not when assessed by means of DHEA (61). Puberty was a predictor of negligible relevance in distinguishing between different depressive symptom trajectories in young adolescents (57), indicating that variations in pubertal timing may not be specific enough to predict individual trajectories of psychopathology. Notably, a prediction model that used electronic primary care records did not find puberty to be a significant predictor, likely because the authors conceptualized puberty as a code of either early or late pubertal timing added to the record by primary care staff (58). This likely required pronounced deviations from the average and points to the importance of capturing variations within a healthy range that may already affect social and psychological factors.

Only a few studies have looked at puberty as a predictor of other diagnoses. One found that puberty, whether measured with the Pubertal Development Scale (59) or captured by steroid hormones (60), was less relevant than other factors in predicting a diagnosis of BPD. Only one study explored its role in relation to psychosis risk (62), but the findings did not allow for any conclusion to be drawn about its utility as a predictor. Overall, puberty represents a potentially important feature in the individualized prediction of adolescent mental health, particularly in the prediction of internalizing symptoms, but its predictive utility could well depend on how pubertal features are conceptualized and measured. While there are many potential barriers to assessing puberty correctly, the inclusion of a self-report measure of physical development could represent a relatively easy-to-implement but very important assessment to investigate as a potential predictor of mental health difficulties in young people.

Notably, all studies that identified puberty as a significant predictor of poor mental health assessed visible physical pubertal changes, while studies that assessed hormonal and proteomic pubertal features did not find them to be important predictors (60, 61, 62). Similarly, 3 studies compared puberty measures and reported stronger associations between physically visible pubertal features rather than hormonal ones and poor mental health (18,48,52). Two studies that did find significant effects for early pubertal hormone exposure increased their discriminative power by restricting the analysis to children with low and high androgen levels (17,44). Low DHEA levels have been associated with stress-related psychopathology (35,56) in adults, which may interfere with finding a potential effect of early initiation of puberty, as would be reflected by relatively high DHEA levels at an early age. Overall, exposure to rising steroid hormones at an early age may have a weaker, and therefore harder to detect, association with mental health difficulties compared with the early development of visible physical characteristics (70). This highlights the relevance of possible psychosocial mechanisms that can be captured by timing the appearance of visible characteristics, such as difficulties in adapting to looking different from same-aged peers, attracting unwanted attention, or experiencing bullying and loneliness, which in turn could affect someone’s mental health. Preventive programs could therefore aim to address social stressors such as bullying and social pressure, for example by implementing puberty education programs in schools.

All studies identified by the 2 searches investigated only pubertal timing features, and no study examined the relationship between pubertal tempo, brain structure, and mental health problems or the predictive utility of pubertal tempo for adolescent mental health outcomes. Given that variations in pubertal tempo have been associated with unfavorable mental health outcomes (5,22) and altered developmental trajectories in brain structure (24), we believe that there is a need for future research that investigates both pubertal timing and tempo. The existing literature does not allow any conclusions to be drawn regarding the specific mechanisms by which the role of puberty and brain structure may differ across mental health disorders because we found too few studies with comparable methodology and outcomes to distinguish between mental health domains. However, given that most adult mental health disorders emerge in adolescence (3), it is still important to investigate the general relationship between puberty and brain structure and their emergence.

Despite a growing body of research investigating the dynamics between puberty, brain structure, and adolescent mental health, to date there is no consistent evidence that explains how brain structure may be related to pubertal and psychopathological processes or whether structural brain changes function as a potential mediator of the link between early puberty and mental health problems. In contrast, evidence is relatively consistent in suggesting that early pubertal timing is a risk factor for mental health problems in young people. In most prediction models, puberty measured by means of physically observable features was relevant for predicting mental health difficulties in adolescence. Together, these insights emphasize the relevance of considering puberty in the prediction of mental health outcomes in young people, particularly by means of assessing observable physical features. It is important that researchers consider the evaluation of puberty staging when designing studies in adolescents and include puberty in individualized prediction models of poor mental health in this age group. Lastly, because physically visible pubertal features seem to be more predictive than hormonal ones, the potential relevance of social processes should also be assessed in future work because it may represent an important target for preventive and intervention strategies during this vulnerable time in life.
