# Supplementary material for: The Dynamic Interplay Between Puberty and Structural Brain Development as a Predictor of Mental Health Difficulties in Adolescence: A Systematic Review
Source: Biol Psychiatry. 2024 Oct 1;96(7):585–603. doi: 10.1016/j.biopsych.2024.06.012 (PMC11794195; doi:10.1016/j.biopsych.2024.06.012)
Supplement: Supplementary Material [file mmc1.pdf]

SUPPLEMENTARY INFORMATION

The Dynamic Interplay Between Puberty and Structural Brain Development as a Predictor of Mental Health Difficulties in Adolescence: A Systematic Review

Kretzer *et al.*

Supplementary Methods

This review included two searches guided by the Preferred Reporting Items for Systematic Reviews and Meta-Analysis (PRISMA) 2020 statement (1). We present (1) a systematic review of research on the relationship between puberty, brain structure, and psychopathology in adolescence, and (2) a review of current risk calculation tools that, among others, included pubertal features to predict mental health outcomes in young people. The first search was preregistered on PROSPERO (2).

Search strategies

We searched PubMed, Web of Science, and PsycINFO for publications up until the 1st of February 2024. The first search included publications from when first available. In contrast, the search for prediction models was limited to papers published after the 1st of January 2021, thus updating searches on this topic already performed in existing reviews on prediction models for mental health outcomes (3-5). These were screened to identify potentially relevant papers published before 2021.

Search strings

Puberty, brain structure, mental health

(mental health OR mental illness OR mental disorder OR psychopath* OR psychological distress OR psychiat* OR internali* OR externali* OR behavio* problems OR behavio* difficulties OR disorgan* OR conduct* OR obsess* compuls* OR mood disorder OR affective disorder* OR affective symptom* OR depress* OR anxi* OR psychosis OR schizophren*) AND (puberty OR pubertal OR tanner stage OR menarche OR sexual maturation) AND (MRI OR magnetic resonance imaging OR DTI OR diffusion tensor imaging OR neuroimaging).

Prediction modelling

(((prognostic scor* OR predict* model* OR risk assessment OR risk score OR risk predict* OR risk calculat* OR risk model* OR machine learn*) AND (score OR scoring OR index OR model* OR predict*) AND (develop* OR derivat* OR valid* OR predict* OR discriminat* OR accura* OR reliab*)) AND (Child OR Children OR Teen* OR Adolesc* OR Youth OR Young OR Juvenile) AND (mental health OR mental illness OR mental disorder OR psychopath* OR psychological distress OR psychiat* OR internali* OR externali* OR behavio* problems OR behavio* difficulties OR disorgan* OR conduct* OR obsess* compuls* OR mood disorder OR affective disorder* OR affective symptom* OR depress* OR anxi* OR psychosis OR schizophren*)).

The first search targeted studies that specifically assessed and provided a measure of puberty and therefore did not include search terms related to adolescence only. In contrast, since many prediction modelling studies only include a full list of predictors briefly in the text or in the supplementary material, a search string that only includes puberty would likely miss potentially relevant papers. For the second search, we therefore opted to include terms referring to adolescence, to ensure relevant studies predicting mental health in young people were identified.

Screening and data extraction

The first search identified 1430 records. Search results were downloaded, and 454 duplicates were removed based on doi, PubMed ID, title, and authors. After de-duplication, authors SK and AJL double-screened 976 unique records, and 38 articles were identified as potentially relevant by either author and included in the full-text screening. Disagreements during any phase were resolved in discussion with a 3rd reviewer to reach a consensus where necessary. Data were extracted including author names, year of publication, type of study design, study population or name of cohort, sample size, age range, sex distribution, ethnicities, measures

of puberty, mental health outcomes, neuroimaging outcomes and software used, and key findings.

The review of prediction models mirrored the first search methods, although single screening was employed (SK). A random sample of 10% of records was double screened (AJL) with 97% agreement and no missed studies. The search identified a total of 2036 records, and 405 duplicates were removed, yielding 1631 unique records to screen. A total of 147 articles were marked as potentially relevant for full-text screening. Data were extracted including author names, year of publication, predicted mental health outcomes, number of categories the model discriminated, study population or name of cohort, sample size, age range, predictor types, puberty measures, model type, validation techniques, model classification performance, and performance of puberty.

Inclusion and exclusion criteria

Both searches targeted studies that included participants of any age provided puberty was assessed, and we did not apply any age restriction *a priori*. We only included studies that specifically evaluated pubertal stage for each individual, and that either included a tight age range or controlled for age in the analyses. Further, we aimed at including all studies measuring pubertal stage, conceptualizing pubertal timing and/or tempo, without any specifications on the aspects of puberty investigated.

For the first search, we included case-control, cohort, and cross-sectional studies published with full-text available and written in English. Studies with young people and with participants with and without mental health difficulties were included. The following variables were specified as necessary for inclusion:

1. **Pubertal status** assessed by means of evaluation of hormone levels, parent- and/or self-report questionnaires, and/or physical examination.
2. **Brain structure** evaluated with MRI, including structural and diffusion-weighted imaging.
3. **Mental health problems** assessed by means of interviews, parent- and/or self-report questionnaires, and including internalizing and externalizing symptom domains, behavioral difficulties, and psychotic experiences.

For the prediction modelling search, studies were included that used prediction modelling methods to discriminate mental health outcomes in young people. For the title and abstract screening, the model containing puberty was no inclusion criterium, as it often required more thorough, full-text, screening to identify whether puberty was included as a predictor.

For both searches, animal research, and studies on populations diagnosed with puberty-related, chromosomal/genetic, endocrine, metabolic, and/or neurodevelopmental disorders were excluded. Reasons for exclusion after screening the full-text articles from the systematic search included: puberty measures used to select or match participants were not included in the analyses, puberty modelled as a covariate of no interest or as a common age-puberty factor, the study did not investigate a relationship between puberty, mental health, and structural MRI, the study did not include mental health measures or structural neuroimaging measures, and the study was a pilot investigation corresponding to an included study (described in the text). For the prediction modelling search, the main reason for exclusion after the full-text screening was puberty not being considered among model predictors, not enough information on puberty collected in the validation samples, the paper was a study protocol or was withdrawn, and the paper did not predict mental health outcomes.

Quality assessment

Quality of the studies for the systematic review was based on selection, comparability, and outcome using the Newcastle–Ottawa scales for cross-sectional and cohort studies (6).

Scores of five or six indicate satisfactory study quality, and scores of seven or more reflect good study quality (7). Scores ranged from seven to nine, indicating good quality for all studies included in the systematic review (Table S1 & S2). For studies selected by the prediction modelling search, the Prediction model risk of bias assessment tool (PROBAST) (8) was used for the risk of bias assessment and risk of bias ranged from low (n=7) to unclear (n=6) (Table S3).

Supplementary Results

Search 1: Study Characteristics

The 17 included publications were based on 13 independent datasets, with most study sites located in the United States (N=9). The remaining sites were Australia (N=2), Japan (N=1), and one study with multiple sites across Europe. Participant age ranged from 6 to 19 years and sample sizes were between 39 and 10,167 participants. Nine studies were population-based, five investigated participants with a psychiatric diagnosis, and three selected participants based on specific characteristics (described below). All studies were rated good quality (Supplementary Tables S2 and S3).

The puberty measures used most frequently were the Pubertal Development Scale (PDS, N=9) and the Tanner stages (N=8), whilst six studies evaluated hormones to capture progression of puberty. Some studies combined different measures (e.g., PDS and pubertal hormones) and reporting forms (e.g., parent- and self-report), with most using parent- and self-report of physically visible puberty characteristics, and two using clinician ratings. Studies evaluated a range of structural neuroimaging morphometric parameters, including cortical thickness and surface area, and white matter structure, with most studies investigating cortical and subcortical volumes. The most widely used measures to assess mental health were the Child Behavior Checklist (CBCL) and different versions of the Kiddie Schedule for Affective and Schizophrenic Disorders (K-SADS), relying on self- or parent-/ guardian-report, or combining both reporting forms.

Search 1: Do puberty and early psychopathology explain brain structural alterations? – Detailed Description

Two studies in this area focused on subcortical structures in youths with a history of trauma and one on youths with bipolar disorder. One longitudinal study found that pre- and early pubertal children with a diagnosis of post-traumatic stress disorder (PTSD) showed right amygdala volume increases over time, while pubertally more mature youths with PTSD showed a decrease, with the controls not showing any significant puberty-related volume change (9). In contrast, a second cross-sectional investigation comparing children with a diagnosis of PTSD and matching controls found no volumetric differences in pituitary volume in both a pilot report (10) and in the full sample (11). A post-hoc subgroup analysis however revealed that pubertal and post-pubertal, but not pre-pubertal participants with a diagnosis of PTSD had larger pituitary volume than controls (11). In a third cross-sectional study, Ahn *et al.* (12) found that pre-pubertal children with a diagnosis of bipolar disorder had significantly larger nucleus accumbens (NAc) volume than both the pubertal children with the same diagnosis and the controls.

All three studies evaluating white matter structure were cross-sectional. Rogers *et al.* (13) found that adolescents with a diagnosis of conduct disorder, independently of pubertal stage, all exhibited higher axial diffusivity (AD) in the corpus callosum, and lower radial diffusivity (RD) and mean diffusivity (MD) in the anterior thalamic radiation compared to a control group. In a sample of adolescent Latina girls, pre-pubertal girls (but not those at more advanced pubertal stages) showed an association between anxiety symptoms and lower cingulum fractional anisotropy (FA) (14). Lastly, Santos *et al.* (15) investigated adolescent anxiety around one-week post-concussion. Concussion can cause injuries in brain microstructure that are associated with internalizing, somatic, and cognitive problems, which persist in about a third of individuals, particularly in adolescents. Compared to controls, adolescents with concussion showed higher anxiety (but not depressive) symptoms in association with lower neurite density indices in white matter tracts responsible for emotion regulation, with greater effects in adolescents at more advanced pubertal stages (15).

Search 1: How do puberty and brain development explain the emergence of mental illness in young people? – Detailed Description

Ten studies investigated whether puberty and brain structure create a sensitive window for the development of psychopathology, with eight exploring whether brain structure mediates the link between puberty and mental health difficulties, one study testing a pubertal stage by brain structure interaction, and one investigating pubertal stage as covariate . Studies are presented below according to the pubertal parameters investigated, including pubertal hormones, observable physical development, or both.

Two cross-sectional studies evaluated children with relatively low and high androgen levels. In the first study, a larger pituitary volume was found to mediate the relationship between early pubertal timing, indicated by relatively high DHEA and DHEA-Sulfate (DHEA-S) levels, and increased social anxiety symptoms (16). Similarly, in a second investigation, larger right hippocampal volume acted as a mediator between early pubertal timing, reflected in relatively high testosterone levels, and higher depressive symptoms, but only in females (17). This evidence points to a link between early exposure to pubertal hormones and internalizing symptoms, mediated by structures sensitive to these hormones such as the pituitary and the hippocampus.

Five studies used measures of observable pubertal development with parent- and child-report questionnaires, and two of these reported on brain measures that mediated the link between early puberty and mental illness. Two papers used the same Australian cohort, including n=155 children (age 12). In the first study, a larger pituitary volume predicted an increase in anxiety symptoms after around 2½ years and was associated with more advanced pubertal maturation. However, pubertal maturation did not predict higher anxiety symptoms (18). The second study investigated a mediation model, showing that early pubertal timing predicted higher depressive symptoms two and half years later, and this link was indeed mediated by larger pituitary volume (19). In a Japanese cohort of 152 children (age 6-18), the link between early pubertal timing and more behavioral and emotional difficulties two years later was found to be mediated by smaller subgenual anterior cingulate cortex volume, but only in females (20).

Two studies on the large US-based Adolescent Brain Cognitive Development (ABCD) cohort found no mediating or interacting effects of structural brain measures. Specifically, MacSweeney *et al.* (21) found that early pubertal timing was associated with more depressive symptoms after two years, with a stronger effect in girls. Here, although a smaller NAc volume was associated with both early pubertal timing and more depressive symptoms, this did not significantly mediate their relationship, nor did the other brain measures investigated (Table 2). In this sample Wiglesworth *et al.* (22) showed that more advanced pubertal maturation at baseline predicted higher internalizing symptoms, but not suicidal ideation, two years later. These authors found that cortical thickness was not associated with mental health outcomes, and reported no significant three-way interactions between puberty, cortical thickness, and sex in predicting internalizing symptoms or suicidal ideation.

Finally, three studies investigated both hormonal and physically visible pubertal measures. Two longitudinal studies investigated pubertal hormones and physician-rated pubertal stage, covariance between whole-brain cortical thickness and amygdala volume, and cognitive, behavioral, and psychological outcomes (23, 24). Negative medial prefrontal cortex-amygdala covariance mediated the link between higher testosterone levels suggestive of more advanced pubertal development and higher levels of aggressive behavior (23). Neither study found a significant effect of puberty measures and cortico-amygdala covariance on internalizing symptoms (23, 24). However, the authors stated that the null effects might have been due to the low variance in internalizing symptoms in their sample (24). Dehestani *et al.* (25) modelled

pubertal timing based on steroid hormones and parent-reported physical maturation and computed structural brain maturation relative to chronological age (Table 2). Early pubertal timing was related to more advanced brain age, and to higher mental health problems, but more advanced brain age did not predict mental health problems, nor did it mediate the relationship between early puberty and mental health difficulties.

One cross-sectional study investigated salivary DHEA, pituitary volume, and trauma-related mental health in nine- to 17-year-olds and tested two models with different outcomes: (1) pituitary volume and (2) anxiety, depressive, or PTSD symptoms. First, lower levels of salivary DHEA mediated the association between higher trauma-related anxiety and smaller anterior pituitary volume, whereas higher anxiety symptoms were related to larger posterior pituitary volume. Second, higher DHEA was related to larger anterior pituitary volume, whilst there were no direct effects of DHEA on either symptom domain, and no mediation by pituitary volumes (26).

Search 2: Study Characteristics

Of the 13 prediction modelling studies identified, six used cross-sectional, and seven longitudinal datasets. Most models were developed with samples from the general population (three from the ABCD cohort), and six models included young people at high risk for, or with, a diagnosis of a psychiatric illness, of which four also included a control group. The overall age range of the studies identified was 3 to 24 years (note that age at inclusion was considerably prior to puberty in long-term longitudinal studies). Sample sizes ranged from 374 to 67,321 cases and up to 192,135 controls (in a study that used electronic primary care records). Risk of bias for the included models was low (n=7) to unclear (n=6) (see Supplementary Table S3). All outcomes were categorical, with twelve models classifying binary outcomes, and just one study predicting a multinomial outcome (membership of symptom trajectory groups, n=4). Most models predicted internalizing symptom status and included self- and parent-report of visible pubertal development characteristics, while four studies included pubertal biomarkers. Most studies used linear regression-based techniques and most internal validation techniques.

Search 2: How do prediction models for adolescent mental health outcomes perform, and are pubertal timing features important for prediction above and beyond other features? – Detailed Description

Rothenberg *et al.* (27) included puberty in their predictive model and provided quantitative information on its individual predictive utility. The researchers used machine learning on a wide range of individual and environmental risk factors (n=79) assessed in 10-year-old children to predict the subsequent onset of internalizing and externalizing symptoms at ages 13 and 17. The models were used to identify the most important predictors and showed overfitting (Matthew Correlation Coefficients (MCCs)=0.65-0.88 development, 0.23-0.46 validation dataset). Puberty assessed with PDS was among the top 15 most important predictors of internalizing symptoms at age 13, but not of externalizing symptoms or symptoms at age 17. Similarly, Su *et al.* (28) developed random forest machine learning models with sociodemographic, pubertal, physical and mental health, parenting, social and school-related features assessed at age 14 to 15 years to predict self-harm and suicide attempts at age 16 to 17 years. The models performed well in the validation sample (AUROC=0.74 self-harm; AUROC=0.72 suicide attempt) and self-reported puberty was the fifth most important predictor for predicting self-harm, while not being among the top 20 features for predicting suicide attempts. This suggests that variability in puberty is a relatively important predictor for internalizing symptoms in early adolescence (age 13) and for self-harm in later adolescence, but might be less relevant for later onset of internalizing symptoms (age 17) and for predicting suicide attempts (27, 28).

Four studies listed puberty as a predictor in their final models but did not provide a breakdown of the statistics on the importance of puberty as a feature. Hawes *et al.* (29) used predictors assessed at multiple ages (3, 6, 9, 12), including hormonal and physically visible puberty features, to predict depression and anxiety at age 15. The model yielded adequate performances (depression AUROC=0.599-0.751; AUROC=0.621-0.812 anxiety). Based on a prior canonical correlation analysis, PDS assessed at ages 9 and 12 was selected as a significant predictor (29), suggesting that atypical pubertal timing early in adolescence might be predictive of depression and anxiety symptoms at age 15.

The following three studies investigated physically visible puberty features using the PDS. Ho *et al.* (30) predicted a diagnosis of depression in ABCD children (age 8-11) at first assessment, and of its prognosis at 1-year follow-up, using machine learning models with pubertal, demographic, familial risk, circadian, and neuroimaging features. Performance was superior in diagnostic prediction (minimizing mean absolute error (MAE)=3.757-3.761) and adequate in prognostic prediction (MAE=4.255-4.262) (30). A logistic regression model on the IMAGEN cohort successfully included age 14 pubertal, clinical, cognitive, environmental and neuroimaging features to predict the onset of depressive symptoms at ages 16 and 19 (AUROC=0.7-0.72, SD=0.07-0.1 development; AUROC=0.68-0.72 validation) (31). van Velzen *et al.* (32) used penalized logistic regression models with pubertal, physical, social, environmental, clinical psychiatric, cognitive, genetic, and task-based fMRI features predictors to distinguish ABCD participants (age 9-11) with and without suicidal thoughts and behaviors and psychiatric diagnoses. The models successfully distinguished children with suicidal thoughts and behaviors from children without these characteristics (AUROC=0.80-0.81), and children with a psychiatric diagnosis from children without a diagnosis (AUROC=0.71-0.77). However, the models did not distinguish children with suicidal ideation from those with suicide attempts (AUROC=0.49-0.58). Together, the results indicate that puberty could be relevant to the prediction of psychiatric diagnoses, and is relevant for predicting depressive and anxiety symptoms, and suicidal thoughts and behaviors in adolescence, but possibly not to the discrimination between the last two.

Four studies investigated physically observable pubertal measures but did not select them as a feature for inclusion in the final prediction model. First, de Lacy *et al.* (33) compared different machine learning models with a wide range of features (n=160) to predict cases with a psychiatric diagnosis (either anxiety, depression, disruptive behaviors, PTSD, or attention deficit) in children and adolescents presenting with behavioral problems. The best performing models discriminated well cases with psychiatric diagnose from controls (AUROC ≥ 0.94), but puberty was not among the top one to six predictors. Second, Xiang *et al.* (34) used k-nearest neighbor and tree-based machine learning models to classify children from the ABCD cohort (age 9-12) into four depressive symptom trajectory groups. Here, individual symptom development over 2 years was clustered into persistently low, persistently high, decreasing, and increasing trajectories. The model's cross-validated area under the receiving operating characteristic (AUROC) was 0.77-0.9, indicating good performance. However, recursive feature elimination did not identify puberty as one the 11 most important features that distinguished the trajectory groups (34), reflecting negligible relevance. A study by Nichols *et al.* (35) used primary care record data to predict a diagnosis of depression in adolescents and young adults (age 15-24). The final logistic regression model performed well (AUROC=0.70-0.72), which was recently replicated (36), but puberty was removed as predictor in the backward stepwise process, as it was not significant. Of note, in this study pubertal features were simply captured by a code that indicated early/late puberty as being present in the medical record, which was true for only 0.2% of records. This suggests that a definition of early/late puberty timing can only capture extreme, potentially physiologically relevant variation rather than variation within the normal range. Lastly, Van Meter *et al.* (37) aimed to validate a previously developed model to predict a diagnosis of bipolar disorder in a high-risk sample of children (age 6 to 12). Cox regression adequately predicted the emergence of

bipolar disorder over two years (AUROC=0.67, 95%CI=0.61;0.72). Here puberty was not included in the original model but added in the validation approach to investigate its predictive value, but it was not found to be a significant predictor.

Three prediction modelling studies assessed puberty with hormonal or proteomic features that were not judged as predictive for the final model. Wu *et al.* (38) recruited 10- to 18-year-olds with a diagnosis of depression or bipolar disorder, and matched controls. The binary logistic regression performed well in predicting a diagnosis of bipolar disorder (AUROC=0.785 development; AUROC=0.714 validation sample) but it did not include measures of pubertal hormones. Although testosterone levels were different between diagnostic groups, testosterone was not selected in the multivariable prediction model (forward stepwise regression), indicating minor utility for prediction. Mürner-Lavanchy *et al.* (39) used machine learning with seven biological markers to predict whether adolescents seeking help at a psychiatric outpatient clinic and controls had engaged in non-suicidal self-injury in the last year. The models performed fairly (AUROC= 0.67-0.7), but DHEA, which could reflect pubertal progression, was not among the top three predictors. Lastly, a machine learning model using mainly proteomic features predicted transition to psychosis in high-risk participants and participants not at risk for psychosis (age 12 to 18) from two large cohorts based across Europe and the UK. The model yielded excellent prediction of psychosis transition (model with 10 most predictive proteins AUROC=0.99 development; AUROC=0.92 validation sample) (40). Sex hormone binding globulin (SHBG), which may be associated with precocious or early puberty when low, was among the proteins of interest (41). However, based on its predictive performance, SHBG was not selected among the 10 most relevant proteins. Given the limited evidence on SHGB function in puberty, and the possibility that this could reflect reduced insulin sensitivity rather than pubertal processes (42), its potential role remains uncertain.

Supplementary Table S1. Quality of cross-sectional studies according to the Newcastle–Ottawa scale

**Study**	**Selection (max 5)**				**Comparability (max 2)**	**Outcome (max 3)**		**Total score**
	**Representative sample**	**Sample size**	**Comparable groups**	**Exposure/ Risk factor**	**Control variables**	**Assessment**	**Statistical tests**	**Max 10**
Ahn *et al.* (12)	*		*	**	**	**		8
Dehestani *et al.* (25)	*	*	*	**	*	**	*	9
Ellis *et al.* (17)			*	**	**	**	*	8
Glenn *et al.* (14)	*		*	**	*	**		7
Murray *et al.* (16)			*	**	**	**	*	8
Picci *et al.* (26)	*	*	*	**	**	**		9
Rogers *et al.* (13)	*	*	*	**	**	**		9
Santos *et al.* (15)	*		*	**	**	**		8
Thomas and De Bellis (11)	*	*	*	**	*	**	*	9

Supplementary Table S2. Quality of cohort studies according to the Newcastle–Ottawa scales

Study	Selection (max 4)				Comparability (max 2)	Outcome (max 3)			Total score
	Representative sample	Comparable groups	Exposure/ Risk factor	Outcome (Baseline measurement)	Control variables	Assessment	Adequacy of follow-up length	Adequacy of follow-up sample	Max 9
MacSweeney *et al.* (21)	*	*	*		**		*	*	7
Nguyen *et al.* (23)	*	*	*	*	**		*	*	8
Nguyen *et al.* (24)	*	*	*	*	**		*	*	8
Okada *et al.* (20)	*	*	*	*	**		*	*	8
Weems *et al.* (9)	*		*		**	*	*	*	7
Whittle *et al.* (19)	*	*	*	*	**	*	*	*	9
Wiglesworth *et al.* (22)	*	*	*	*	**	*	*	*	9
Zipursky *et al.* (18)	*	*	*	*	**	*	*	*	9

Supplementary Table S3. Risk of bias of prediction modelling studies according to PROBAST

**Study**	**Domain**				**Overall rating**
	**Participants**	**Predictors**	**Outcome**	**Analysis**	
de Lacy *et al.* (33)	+	+	+	+	+
Hawes *et al.* (29)	+	+	+	?	?
Ho *et al.* (30)	?	+	+	?	?
Mongan *et al.* (40)	+	+	+	+	+
Mürner-Lavanchy *et al.* (39)	+	+	+	+	+
Nichols *et al.* (35)	+	+	+	?	?
Rothenberg *et al.* (27)	+	+	+	+	+
Su *et al.* (28)	+	+	+	+	+
Toenders *et al.* (31)	+	+	+	+	+
Van Meter *et al.* (37)	+	+	+	?	?
van Velzen *et al.* (32)	+	+	+	?	?
Wu *et al.* (38)	+	+	+	?	?
Xiang *et al.* (34)	+	+	+	+	+

Supplementary References

1. Page MJ, McKenzie JE, Bossuyt PM, Boutron I, Hoffmann TC, Mulrow CD, et al. (2021): Declaración PRISMA 2020: una guía actualizada para la publicación de revisiones sistemáticas. *Revista Española de Cardiología*. 74:790-799.

2. Kretzer SL, Andrew; Dazzan, Paola (2022): A systematic review of the dynamics between puberty, structural brain alterations and mental health difficulties in adolescence. PROSPERO.

3. Senior M, Fanshawe T, Fazel M, Fazel S (2021): Prediction models for child and adolescent mental health: A systematic review of methodology and reporting in recent research. *JCPP advances*. 1:e12034.

4. Salazar de Pablo G, Studerus E, Vaquerizo-Serrano J, Irving J, Catalan A, Oliver D, et al. (2021): Implementing precision psychiatry: a systematic review of individualized prediction models for clinical practice. *Schizophrenia bulletin*. 47:284-297.

5. Shatte AB, Hutchinson DM, Teague SJ (2019): Machine learning in mental health: a scoping review of methods and applications. *Psychological medicine*. 49:1426-1448.

6. Madhavan A, LaGorio L, Crary M, Dahl W, Carnaby G (2016): Prevalence of and risk factors for dysphagia in the community dwelling elderly: a systematic review. *The journal of nutrition, health & aging*. 20:806-815.

7. Herzog R, Álvarez-Pasquin M, Díaz C, Del Barrio JL, Estrada JM, Gil Á (2013): Are healthcare workers' intentions to vaccinate related to their knowledge, beliefs and attitudes? A systematic review. *BMC public health*. 13:1-17.

8. Wolff RF, Moons KG, Riley RD, Whiting PF, Westwood M, Collins GS, et al. (2019): PROBAST: a tool to assess the risk of bias and applicability of prediction model studies. *Annals of internal medicine*. 170:51-58.

9. Weems CF, Scott BG, Russell JD, Reiss AL, Carrión VG (2013): Developmental variation in amygdala volumes among children with posttraumatic stress. *Developmental Neuropsychology*. 38:481-495.

10. De Bellis MD, Hall J, Boring AM, Frustaci K, Moritz G (2001): A pilot longitudinal study of hippocampal volumes in pediatric maltreatment-related posttraumatic stress disorder. *Biological psychiatry*. 50:305-309.

11. Thomas LA, De Bellis MD (2004): Pituitary volumes in pediatric maltreatment-related posttraumatic stress disorder. *Biological Psychiatry*. 55:752-758.

12. Ahn MS, Breeze JL, Makris N, Kennedy DN, Hodge SM, Herbert MR, et al. (2007): Anatomic brain magnetic resonance imaging of the basal ganglia in pediatric bipolar disorder. *Journal of Affective Disorders*. 104:147-154.

13. Rogers JC, Gonzalez-Madruga K, Kohls G, Baker RH, Clanton RL, Pauli R, et al. (2019): White matter microstructure in youths with conduct disorder: effects of sex and variation in callous traits. *Journal of the American Academy of Child & Adolescent Psychiatry*. 58:1184-1196.

14. Glenn DE, Merenstein JL, Bennett IJ, Michalska KJ (2022): Anxiety symptoms and puberty interactively predict lower cingulum microstructure in preadolescent Latina girls. *Scientific Reports*. 12:20755.

15. Santos JPL, Kontos AP, Holland CL, Suss Jr SJ, Stiffler RS, Bitzer HB, et al. (2022): The role of puberty and sex on brain structure in adolescents with anxiety following concussion. *Biological psychiatry: cognitive neuroscience and neuroimaging*.

16. Murray CR, Simmons JG, Allen NB, Byrne ML, Mundy LK, Seal ML, et al. (2016): Associations between dehydroepiandrosterone (DHEA) levels, pituitary volume, and social anxiety in children. *Psychoneuroendocrinology*. 64:31-39.

17. Ellis R, Fernandes A, Simmons JG, Mundy L, Patton G, Allen NB, et al. (2019): Relationships between adrenarcheal hormones, hippocampal volumes and depressive symptoms in children. *Psychoneuroendocrinology*. 104:55-63.

18. Zipursky AR, Whittle S, Yücel M, Lorenzetti V, Wood SJ, Lubman DI, et al. (2011): Pituitary volume prospectively predicts internalizing symptoms in adolescence. *Journal of child psychology and psychiatry*. 52:315-323.

19. Whittle S, Yücel M, Lorenzetti V, Byrne ML, Simmons JG, Wood SJ, et al. (2012): Pituitary volume mediates the relationship between pubertal timing and depressive symptoms during adolescence. *Psychoneuroendocrinology*. 37:881-891.

20. Okada N, Yahata N, Koshiyama D, Morita K, Sawada K, Kanata S, et al. (2020): Smaller anterior subgenual cingulate volume mediates the effect of girls' early sexual maturation on negative psychobehavioral outcome. *Neuroimage*. 209:116478.

21. MacSweeney N, Allardyce J, Edmondson-Stait A, Shen X, Casey H, Chan SW, et al. (2023): The role of brain structure in the association between pubertal timing and depression risk in an early adolescent sample (the ABCD Study®): A registered report. *Developmental Cognitive Neuroscience*.101223.

22. Wiglesworth A, Fiecas MB, Xu M, Neher AT, Padilla L, Carosella KA, et al. (2023): Sex and age variations in the impacts of puberty on cortical thickness and associations with internalizing symptoms and suicidal ideation in early adolescence. *Developmental Cognitive Neuroscience*.101195.

23. Nguyen T-V, McCracken JT, Albaugh MD, Botteron KN, Hudziak JJ, Ducharme S (2016): A testosterone-related structural brain phenotype predicts aggressive behavior from childhood to adulthood. *Psychoneuroendocrinology*. 63:109-118.

24. Nguyen TV, Jones SL, Gower T, Lew J, Albaugh MD, Botteron KN, et al. (2019): Age-specific associations between oestradiol, cortico-amygdalar structural covariance, and verbal and spatial skills. *J Neuroendocrinol*. 31:e12698.

25. Dehestani N, Whittle S, Vijayakumar N, Silk TJ (2023): Developmental brain changes during puberty and associations with mental health problems. *Developmental Cognitive Neuroscience*.101227.

26. Picci G, Casagrande CC, Ott LR, Petro NM, Christopher-Hayes NJ, Johnson HJ, et al. (2023): Dehydroepiandrosterone mediates associations between trauma-related symptoms and anterior pituitary volume in children and adolescents. *Human Brain Mapping*. 44:6388-6398.

27. Rothenberg WA, Bizzego A, Esposito G, Lansford JE, Al-Hassan SM, Bacchini D, et al. (2023): Predicting Adolescent Mental Health Outcomes Across Cultures: A Machine Learning Approach. *J Youth Adolesc*. 52:1595-1619.

28. Su R, John JR, Lin P-I (2023): Machine learning-based prediction for self-harm and suicide attempts in adolescents. *Psychiatry research*. 328:115446.

29. Hawes MT, Schwartz HA, Son Y, Klein DN (2022): Predicting adolescent depression and anxiety from multi-wave longitudinal data using machine learning. *Psychol Med*.1-7.

30. Ho TC, Shah R, Mishra J, May AC, Tapert SF (2022): Multi-level predictors of depression symptoms in the Adolescent Brain Cognitive Development (ABCD) study. *Journal of Child Psychology and Psychiatry*. 63:1523-1533.

31. Toenders YJ, Kottaram A, Dinga R, Davey CG, Banaschewski T, Bokde ALW, et al. (2022): Predicting Depression Onset in Young People Based on Clinical, Cognitive, Environmental, and Neurobiological Data. *Biol Psychiatry Cogn Neurosci Neuroimaging*. 7:376-384.

32. van Velzen LS, Toenders YJ, Avila-Parcet A, Dinga R, Rabinowitz JA, Campos AI, et al. (2022): Classification of suicidal thoughts and behaviour in children: results from penalised logistic regression analyses in the Adolescent Brain Cognitive Development study. *Br J Psychiatry*. 220:210-218.

33. de Lacy N, Ramshaw MJ, McCauley E, Kerr KF, Kaufman J, Nathan Kutz J (2023): Predicting individual cases of major adolescent psychiatric conditions with artificial intelligence. *Translational psychiatry*. 13:314.

34. Xiang Q, Chen K, Peng L, Luo J, Jiang J, Chen Y, et al. (2022): Prediction of the trajectories of depressive symptoms among children in the adolescent brain cognitive development (ABCD) study using machine learning approach. *J Affect Disord*. 310:162-171.

35. Nichols L, Ryan R, Connor C, Birchwood M, Marshall T (2018): Derivation of a prediction model for a diagnosis of depression in young adults: a matched case–control study using electronic primary care records. *Early intervention in psychiatry*. 12:444-455.

36. Nickson D, Singmann H, Meyer C, Toro C, Walasek L (2023): Replicability and reproducibility of predictive models for diagnosis of depression among young adults using Electronic Health Records. *Diagnostic and Prognostic Research*. 7:25.

37. Van Meter AR, Hafeman DM, Merranko J, Youngstrom EA, Birmaher BB, Fristad MA, et al. (2021): Generalizing the prediction of bipolar disorder onset across high-risk populations. *Journal of the American Academy of Child & Adolescent Psychiatry*. 60:1010-1019. e1012.

38. Wu X, Niu Z, Zhu Y, Shi Y, Qiu H, Gu W, et al. (2022): Peripheral biomarkers to predict the diagnosis of bipolar disorder from major depressive disorder in adolescents. *European archives of psychiatry and clinical neuroscience*. 272:817-826.

39. Mürner-Lavanchy I, Koenig J, Reichl C, Josi J, Cavelti M, Kaess M (2024): The quest for a biological phenotype of adolescent non-suicidal self-injury: a machine-learning approach. *Translational psychiatry*. 14:56.

40. Mongan D, Föcking M, Healy C, Susai SR, Heurich M, Wynne K, et al. (2021): Development of Proteomic Prediction Models for Transition to Psychotic Disorder in the

Clinical High-Risk State and Psychotic Experiences in Adolescence. *JAMA Psychiatry*. 78:77-90.

41. Sørensen K, Andersson A, Skakkebaek N, Juul A (2007): Serum sex hormone-binding globulin levels in healthy children and girls with precocious puberty before and during gonadotropin-releasing hormone agonist treatment. *The Journal of Clinical Endocrinology & Metabolism*. 92:3189-3196.

42. Aydın B, Winters SJ (2016): Sex hormone-binding globulin in children and adolescents. *Journal of clinical research in pediatric endocrinology*. 8:1.
